# 100 nm AlSb/InAs HEMT for Ultra-Low-Power Consumption, Low-Noise Applications

**DOI:** 10.1155/2014/136340

**Published:** 2014-02-23

**Authors:** Cyrille Gardès, Sonia Bagumako, Ludovic Desplanque, Nicolas Wichmann, Sylvain Bollaert, François Danneville, Xavier Wallart, Yannick Roelens

**Affiliations:** Institut d'Électronique de Microélectronique et de Nanotechnologie (IEMN), UMR CNRS 8520, Université Lille I, BP 60069, 59652 Villeneuve d'Ascq Cedex, France

## Abstract

We report on high frequency (HF) and noise performances of AlSb/InAs high electron mobility transistor (HEMT) with 100 nm gate length at room temperature in low-power regime. Extrinsic cut-off frequencies *f*_*T*_/*f*_max_ of 100/125 GHz together with minimum noise figure NF_min_ = 0.5 dB and associated gain *G*_ass_ = 12 dB at 12 GHz have been obtained at drain bias of only 80 mV, corresponding to 4 mW/mm DC power dissipation. This demonstrates the great ability of AlSb/InAs HEMT for high-frequency operation combined with low-noise performances in ultra-low-power regime.

## 1. Introduction

Though the best high frequency performances are obtained for InAlAs/InGaAs HEMT technology which is more mature [1], AlSb/InAs HEMTs are potentially excellent candidates for low-voltage, low-power consumption operation in the case of high-speed analog and digital applications [2]. AlSb/InAs heterostructures are grown since the 1980s [3, 4], but AlSb/InAs HEMT with noticeable RF figures-of-merit and amplifiers with interesting low-noise performances have only been obtained since the last ten years [5, 6].

The best extrinsic *f*_*T*_ of 303 GHz has been reached for a transistor with 120 nm gate length at drain bias of 0.44 V [7]. The main modifications regarding our previous work [7, 8] lie in an optimization of heterostructure growth conditions [9], no ohmic cap layer [10], and the use of alternative metallic gate stack [11]. With this technology, the highest combination of cut-off frequencies obtained simultaneously for AlSb/InAs HEMTs has recently been shown at *V*_*ds*_ = 360 mV [10], beyond previous *f*_*T*_/*f*_max⁡_ record of 260/280 GHz reported for 100 nm HEMT at *V*_*ds*_ = 400 mV [12]. Cut-off frequencies *f*_*T*_/*f*_max⁡_ of 290/335 GHz were obtained for a 120 nm HEMT. We presently focus on HEMT operation in mobility regime (*V*_*ds*_ = 80 mV) in which we will demonstrate that no impact ionization occurs. In these low drain bias conditions, corresponding to ultra-low-power dissipation, previous works report *f*_*T*_/*f*_max⁡_ of 112/107 GHz for (*V*_*ds*_ = 0.1 V; *P*_DC_ = 4.3 mW/mm) [5] and *f*_*T*_/*f*_max⁡_ of 143/115 GHz at (*V*_*ds*_ = 0.1 V; *P*_DC_ = 9.9 mW/mm) [7]. In this study, we present a full set of characteristics at *V*_*ds*_ = 80 mV regarding DC, HF, and noise performances, extracting RF figures-of-merit, extrinsic and intrinsic parameters, and noise parameters obtained from small-signal equivalent circuit with noise sources.

## 2. Heterostructure and Device Fabrication

### 2.1. Heterostructure

**Figure 1:**
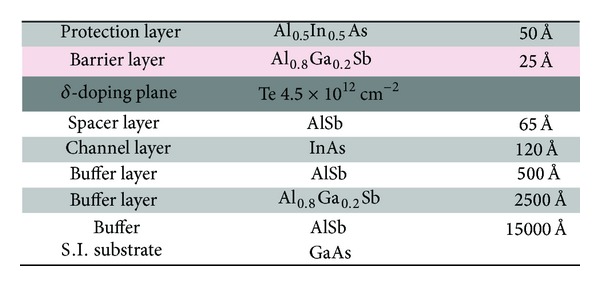
AlSb/InAs heterostructure.

The AlSb/InAs heterostructure was grown by molecular beam epitaxy on 3-inche semi-insulating GaAs substrate. A thick AlSb buffer is used to accommodate the large lattice mismatched between 6.1 Å materials and GaAs substrate. Then, the structure consists of a 120 Å InAs channel, a 65 Å AlSb spacer, a Te *δ*-doping plane, and a composite Schottky barrier with a 25 Å Al_0.8_Ga_0.2_Sb layer and a 50 Å Al_0.5_In_0.5_As layer (Figure 1). The Al_0.5_In_0.5_As layer in the composite Schottky barrier avoids oxidation of Al_0.8_Ga_0.2_Sb with air exposure and acts as a hole barrier [13]. Hall measurements at room temperature exhibit a sheet carrier density of 1.5 × 10^12^ cm^−2^ and electron mobility of 26000 cm²/(Vs), giving sheet resistance of 160 Ω/□.

### 2.2. Device Fabrication

**Figure 2:**
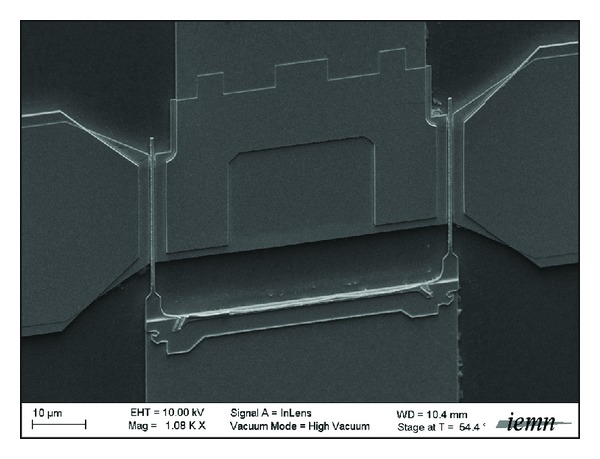
100 nm AlSb/InAs HEMT.

HEMTs fabrication starts with ohmic contact evaporation of Pd/Pt/Au after e-beam lithography, followed by rapid thermal annealing at 275°C. Despite the absence of highly doped cap layer in the heterostructure, contact resistance, obtained by transmission-line model measurements, is still below 0.05 Ω·mm. Schottky T-gate is realized using bilayer resist e-beam lithography process and Mo/Pt/Au metallization. Then, Ti/Au bonding pads are evaporated. Finally, the active area is defined by chemical deep mesa isolation using HF/H_2_O_2_ solution to completely remove the AlSb buffer, leading to air-bridge gate. Device features are a two-finger 100 nm long gate with 2 × 25 *μ*m transistor width (Figure 2). Source-drain spacing is 1.2 *μ*m.

## 3. Static and Dynamic Measurements

**Figure 3:**
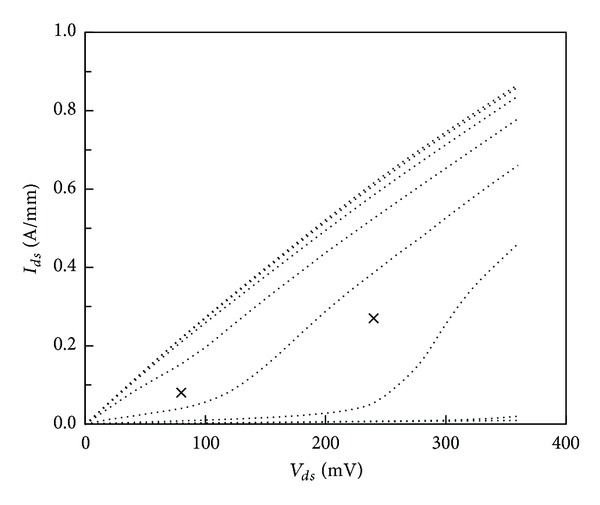
Drain current-voltage characteristic of 100 nm AlSb/InAs HEMT. *V*_*gs*_ is varying from 0 V to −1.4 V with −0.2 V step. (Crosses are polarisation conditions for measurements at peak *f*_*T*_).

Drain current-voltage characteristics are plotted in Figure 3. Pinch-off voltage is −1.0 V. Maximum drain currents are 220 mA/mm and 620 mA/mm for drain bias of 80 mV and 240 mV, respectively. These are similar to our previous results [7, 8] despite the higher sheet resistance of the heterostructure and the higher source-drain spacing in the present device.

**Figure 4:**
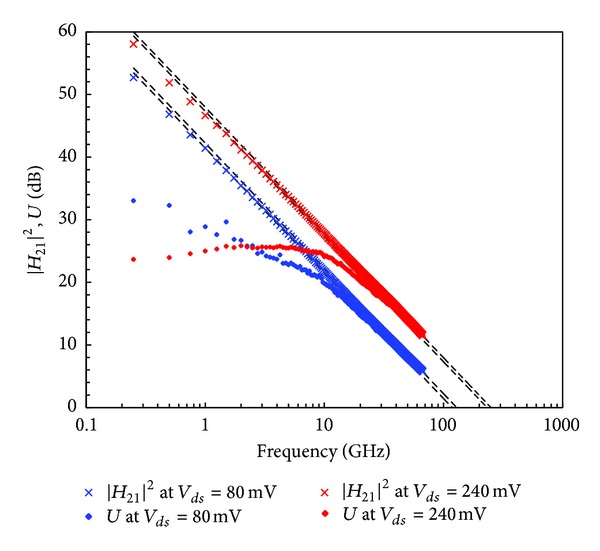
*f*_max⁡_ and *f*_*T*_ extrapolated from Mason's unilateral gain *U* and current gain |*H*_21_|^2^ for *V*_*ds*_ = 80 mV and *V*_*ds*_ = 240 mV.

HF measurement setup consists in a 67 GHz Agilent PNA for *S*-parameters on-wafer measurements and an Agilent HP4142 generator for DC biasing. Extrinsic current gain |*H*_21_|^2^ and unilateral power gain *U* for *V*_*ds*_ = 80 mV and *V*_*ds*_ = 240 mV at peak *f*_*T*_ are presented in Figure 4. Cut-off frequencies (*f*_*T*_, *f*_max⁡_) obtained simultaneously at *V*_*ds*_ = 80 mV are (108 GHz, 129 GHz) for power dissipation *P*_DC_ = 5 mW/mm and (232 GHz, 250 GHz) at *V*_*ds*_ = 240 mV for *P*_DC_ = 60 mW/mm. *P*_DC_ is calculated as *V*_*ds*_ × *I*_*ds*_, with power consumption in the gate being negligible.

**Figure 5:**
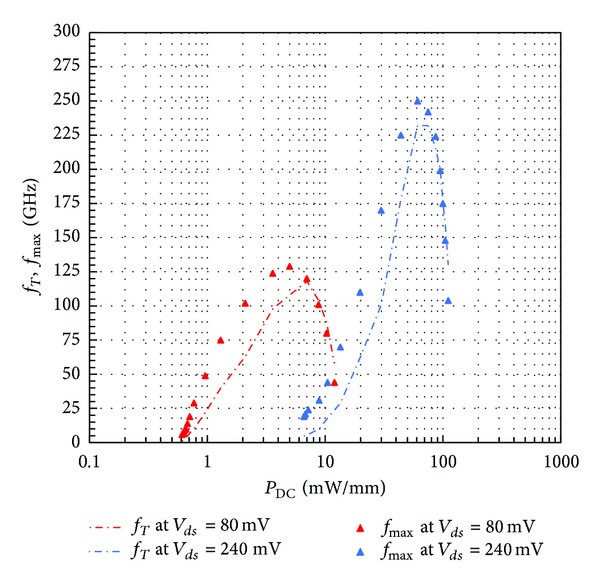
Extrapolated (*f*_*T*_, *f*_max⁡_) plotted as a function of DC power consumption calculated as *V*_*ds*_ × *I*_*ds*_.

In Figure 5, the evolution of extrinsic cut-off frequencies is plotted as a function of *P*_DC_ for *V*_*ds*_ = 80  mV and *V*_*ds*_ = 240  mV. This evidences the ability of AlSb/InAs HEMT for RF performances in low drain bias regime. In fact, (*f*_*T*_, *f*_max⁡_) are (100 GHz, 125 GHz) for *P*_DC_ = 4 mW/mm at *V*_*ds*_ = 80 mV. The DC power consumption at *V*_*ds*_ = 240 mV for reaching the same cut-off frequencies is, respectively, 30 mW/mm and 22 mW/mm. Consequently, to get the same RF performances in more standard drain bias conditions, power consumption must be at least 5 times higher.

**Figure 6:**
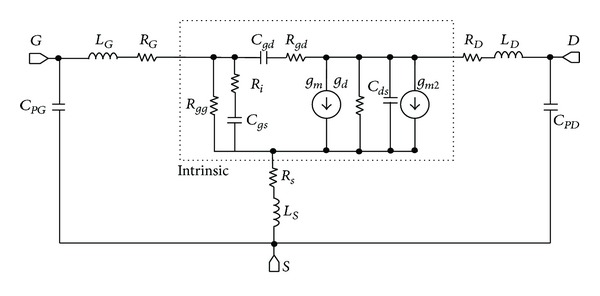
Small-signal equivalent circuit tacking into account gate leakage current (*R*_*gg*_) and impact ionisation (*g*_*m*2_).

Finally, intrinsic and extrinsic parameters have been extracted from the small-signal equivalent circuit (SSEC) presented in Figure 6.

**Figure 7:**
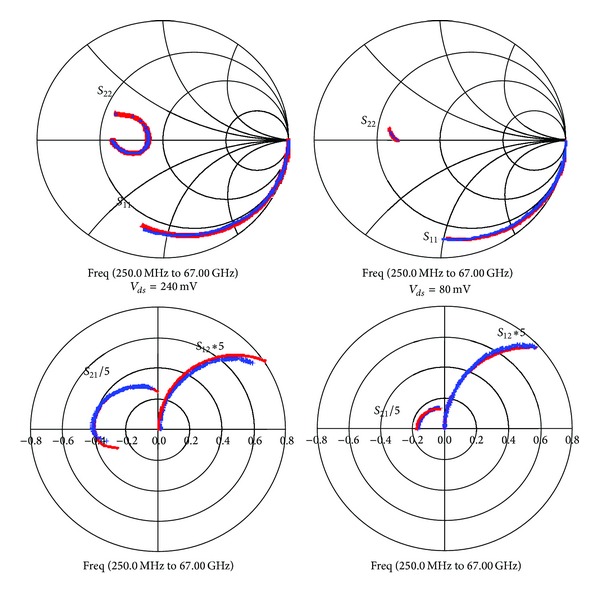
*S*-parameters measured (blue dots) and simulated (red curves) at *V*_*ds*_ = 80 mV and *V*_*ds*_ = 240 mV.

Resistance *R*_*gg*_ parallel to *C*_*gs*_ and current source *g*_*m*2_ parallel to output conductance *g*_*d*_ to account, respectively, for gate leakage current and impact ionization have been added to the classical model. Indeed, there is impact ionization in AlSb/InAs HEMT at high drain bias with an increase of gate current and a typical bell-shape of the *I*_*gs*_-*V*_*gs*_ characteristic [14], which is a signature of impact ionization in DC measurements. With RF characterization, impact ionization results in *S*_22_ parameter evolving from inductive to capacitive behaviour with increasing frequency as can be seen for *V*_*ds*_ = 240 mV in Figure 7. In the literature, this phenomenon in HEMTs has been modelised with a low-pass filter [15]. We prefer to introduce an additional current source *g*_*m*2_ controlled by gate-drain voltage as realized by Isler [16] to account for impact ionization effects. This model allows to perfectly fit scattering parameters at *V*_*ds*_ = 80 mV and *V*_*ds*_ = 240 mV as shown in Figure 7.

Parameters extracted from the SSEC at peak *f*_*T*_ are presented in Table 1. *R*_*gg*_ is much higher at *V*_*ds*_ = 80 mV compared to *V*_*ds*_ = 240 mV, which is relevant of much lower gate leakage current, and *g*_*m*2_ is negligible at *V*_*ds*_ = 80 mV, which stresses that there is no impact ionization at this drain voltage.

## 4. Noise Measurements

**Figure 8:**
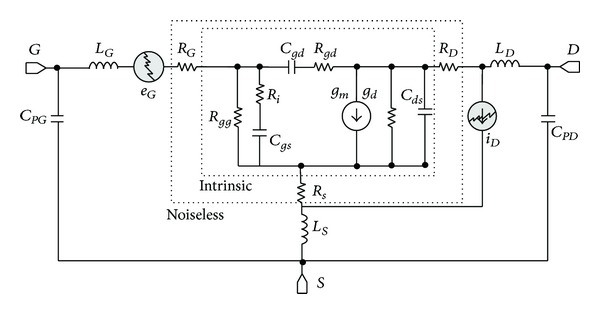
Small-signal equivalent circuit with noise sources for AlSb/InAs HEMT at *V*_*ds*_ = 80 mV.

**Figure 9:**
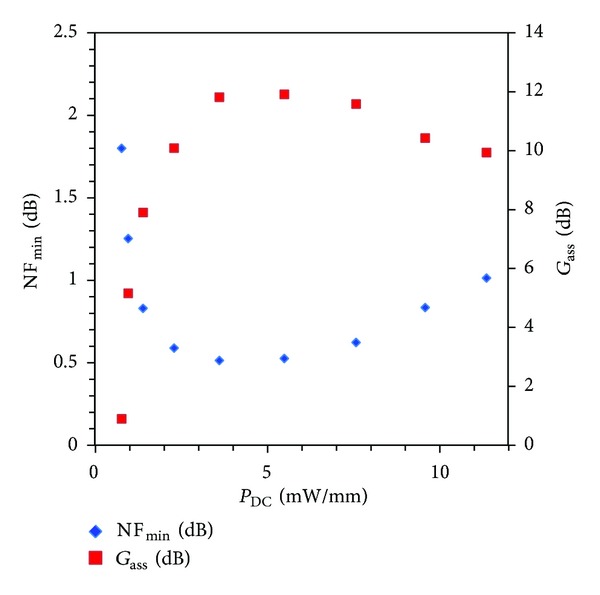
Minimum noise figure NF_min⁡_ and associated gain *G*_ass_  as a function of power consumption at 12 GHz for *V*_*ds*_ = 80 mV.

**Figure 10:**
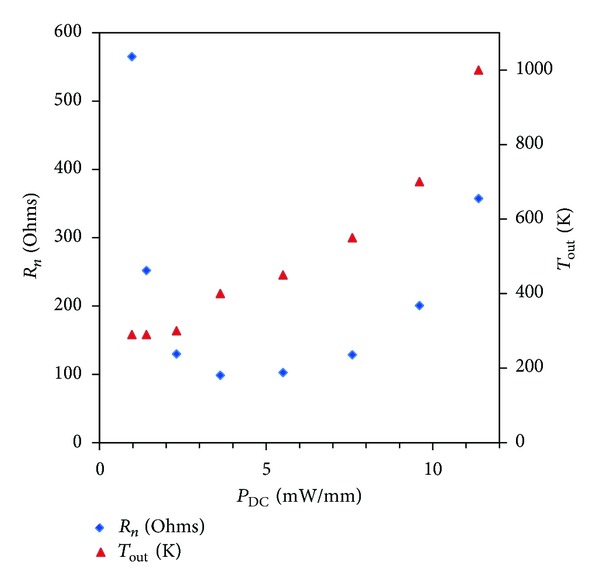
Noise equivalent resistance *R*_*n*_ and output noise temperature *T*_out_ as a function of power consumption at 12 GHz for *V*_*ds*_ = 80 mV.

Regarding low impact ionization occurring at *V*_*ds*_ = 80 mV as shown above with RF wideband measurements, SSEC with noise sources as presented in Figure 8 is used. For the sake of simplicity, there is no current source accounting for impact ionization since extracted value of *g*_*m*2_ at *V*_*ds*_ = 80 mV is negligible. As a consequence, no additional noise source, which should probably be correlated with output noise current or even input noise voltage, is required for extraction of accurate parameters values. We extracted the following noise parameters using *F*_50_ method [17]: minimum noise figure NF_min⁡_, associated gain *G*_ass_, noise equivalent resistance *R*_*n*_, and output noise temperature *T*_out_ at 12 GHz (Figures 9 and 10). NF_min⁡_ is 0.5 dB and *G*_ass_ is 12 dB for 4 mW/mm power dissipation. As a comparison, we should quote results obtained by Ma et al. [5] for 2 × 20 *μ*m HEMT with NF_min⁡_ above 0.5 dB at 12 GHz in the “best bias conditions for minimum noise figure.” The present results should also be compared with similar NF_min⁡_ and *G*_ass_ reported in literature for AlSb/InAs HEMTs but with 50% higher DC power consumption of 6 mW/mm at *V*_*ds*_ = 200 mV [6, 18]. In the present case, at *V*_*ds*_ = 80 mV, NF_min⁡_, and *G*_ass_ are optima for *P*_DC_ = 4 mW/mm and it is important to underline that it would be impossible to reach these noise performances at *V*_*ds*_ = 240 mV with such low-power consumption. Despite an accurate extraction of noise parameters under high drain bias is not done here, the element values would obviously be degraded due to the higher gate voltage required to operate in low-power regime, which would increase shot noise. Then, drain polarization of transistor at *V*_*ds*_ = 80 mV allows an excellent compromise between noise performances and power dissipation.

## 5. Conclusion

In this study, we reported on microwave and noise performances in low-power regime of AlSb/InAs HEMTs with optimized heterostructure. Combined (*f*_*T*_, *f*_max⁡_) of (100 GHz, 125 GHz) have been obtained at *V*_*ds*_ = 80 mV and DC power consumption of 4 mW/mm, performances that cannot be reached at *V*_*ds*_ = 240 mV for such a low power dissipation. A small-signal equivalent circuit was established and demonstrated that impact ionization effects at *V*_*ds*_ = 80 mV are negligible, which is not the case for *V*_*ds*_ = 240 mV. This allowed an accurate extraction of noise parameters thanks to SSEC with noise sources fully reliable in mobility regime. NF_min⁡_ = 0.5 dB and *G*_ass_ = 12 dB have been obtained at 12 GHz for (*V*_*ds*_ = 80 mV; *P*_DC_ = 4 mW/mm). These results exhibit the high suitability of AlSb/InAs HEMTs for combined RF and low-noise performances in ultra-low-power dissipation regime.

## Figures and Tables

**Table 1:** Small-signal equivalent circuit parameters for *V*_*ds*_ = 80 mV and *V*_*ds*_ = 240 mv at peak *f*_*T*_.

*V*_*ds*_ (mV)	*R*_*G*_ (Ω/mm)	*R*_*D*_ (Ω·mm)	*R*_*S*_ (Ω·mm)	*g*_*m*_ (S/mm)	*g*_*d*_ (S/mm)	*C*_*gs*_ (fF/mm)	*C*_*gd*_ (fF/mm)	*g*_*m*2_ (S/mm)	*R*_*gg*_ (k Ω)
80	74	0.11	0.11	0.75	0.73	468	327	0.007	80
240	74	0.11	0.11	1.36	0.72	584	259	1.47	12
